# Chemo-enzymatically prepared lignin nanoparticles for value-added applications

**DOI:** 10.1007/s11274-019-2697-7

**Published:** 2019-07-30

**Authors:** Alexander Henn, Maija-Liisa Mattinen

**Affiliations:** Department of Bioproducts and Biosystems, Bioproduct Chemistry, School of Chemical Engineering, Aalto University, P.O. Box 16300, Aalto, 00076 Espoo, Finland

**Keywords:** Cosmetics, Foods, Functionalization, Lignin, Medicine, Nanoparticle

## Abstract

**Abstract:**

The global need to develop sustainable materials and products from non-fossil raw material is pushing industry to utilize side-streams more efficiently using green processes. Aromatic lignin, the world’s second most abundant biopolymer, has multiple attractive properties which can be exploited in various ways instead of being burnt or used as animal feed. Lignin’s poor water solubility and its highly branched and random structure make it a challenging biopolymer to exploit when developing novel technologies for the preparation of tailored nanobiomaterials for value-added applications. The notable number of scientific publications focusing on the formation and modification of technical lignin in nanoparticulate morphology show that these bottlenecks could be solved using lignin in the form of colloidal particles (CLPs). These particles are very stable at wide pH range (4–11) and easily dispersible in organic solvents after stabilized via cross-linking. Negative hydroxyl groups on the CLP surface enable multiple enzymatic and chemical modifications e.g. via polymerization reactions and surface-coating with positive polymers. This contribution highlights how tailored CLPs could be innovatively exploited in different *the state-of-the-art* applications such as medicine, foods, and cosmetics.

**Graphic abstract:**

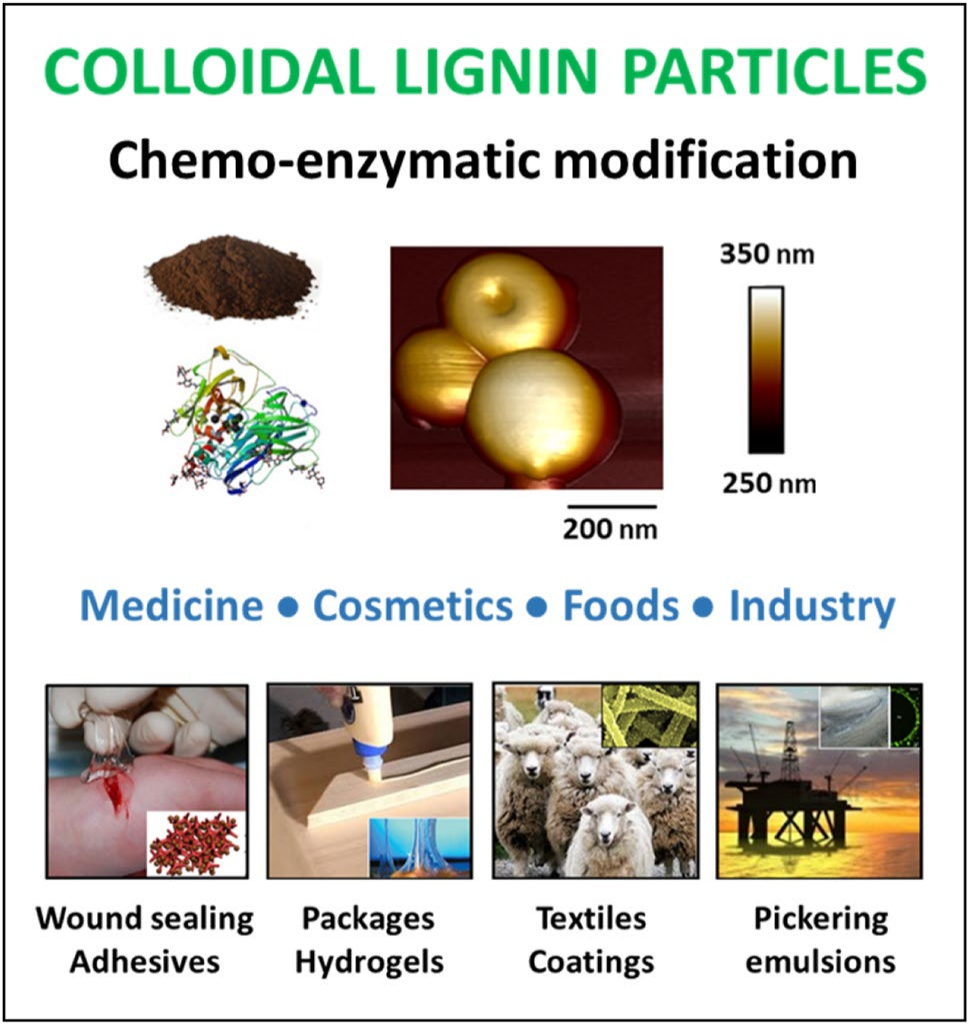

## Introduction

Decades of non-sustainable use of oil-based products in massive scale has resulted in one of the biggest concerns in the modern society, promoting industry and households towards more sustainable lifestyles (Kai et al. 2016). Fossil-based chemicals and polymers are difficult to compete with. Products and materials developed from these raw materials are cheap, customizable and their production is very cost-effective. Increased utilization of bio-based raw materials from the industrial side-streams, development of green technologies and products are global missions (Upton and Kasko 2016; Cao et al. 2018; Solt et al. 2019).

Lignin, the poorly water-soluble, branched and heterogeneously structured aromatic polymer is a challenging by-product to exploit. Despite several attractive properties such as biodegradability, UV-absorptivity, excellent thermal stability, antioxidant and antimicrobial properties, reactivity via radicalization, intense color varying from yellow via red to black, lignin is largely considered as waste from biorefinery, pulp and paper processes (Patil and Argyropoulos 2017; Qian et al. 2014). Since it is mostly burnt for energy or used as animal feed (Beisl et al. 2017; Yang et al. 2016a; Gilca et al. 2015), the need for the development of green chemo-enzymatic lignin valorization technologies is apparent (Ragauskas et al. 2014; Cannatelli and Ragauskas 2016; Husarcíková et al. 2018; Ma et al. 2018). The use of technical lignin in nanoparticulate morphology (Duval and Lawoko 2014; Henn and Mattinen 2019) is an interesting method which could potentially overcome many challenges in the processing regarding the polymer’s heterogeneity and poor water solubility. The number of publications focusing on the preparation and functionalization of CLPs has increased rapidly in recent years which in itself is a demonstration of their usefulness (Zhao et al. 2016; Lievonen et al. 2016; Frangville et al. 2012; Gilca et al. 2015; Qian et al. 2014; Sipponen et al. 2017; Ago et al. 2016, 2017).

CLPs are easily water dispersible and they can be dispersed in organic solvents after stabilized with covalent cross-linking (Nypelö et al. 2015; Mattinen et al. 2018a). The phenol and hydroxyl groups in lignin enable multiple chemical and enzymatic modifications e.g. via radicalization (Patil and Argyropoulos 2017; Liu et al. 2015; Mattinen et al. 2018a, b; Ma et al. 2018) and hence the vast number of potential applications (Norgren and Edlund 2014; Tardy et al. 2018; Figueiredo et al. 2018). Several studies have shown that CLPs are non-cytotoxic in the living cells, with the effect depending notably on the concentration of CLPs and the cell type of the organism (Figueiredo et al. 2017; Tortora et al. 2014; Gilca and Popa 2013). Although scientific research focusing on the exploitation of CLPs is still at an early stage compared e.g. to nanocellulose, many *the state-of-the-art* technologies have already shown their feasibility. In this contribution, the focus is on chemo-enzymatic modification of CLPs to meet requirements of the value-added applications. Furthermore, this review aims to bring novel insights highlighting some of the recent biotechnological developments.

## Preparation of CLPs

There are many simple ways to synthesized CLPs, but one of the first ones was published by Frangville et al. (2012). Nanosized lignin particles were prepared from sulfonated Indulin AT by dissolving lignin in ethylene glycol following precipitation using diluted HCl and dialysis with H_2_O. Addition of glutaraldehyde in the lignin–ethylene glycol solution produced covalently cross-linked CLPs. The initial lignin concentration and the speed of the precipitation had a significant effect on the particle size varying from 200 to 400 nm. The cross-linking slightly improved the stability of the CLPs at neutral pH. The zeta potential was ca. – 37 mV for noncross-linked and ca. – 20 mV for the cross-linked CLPs in pH range 6–9. When lignin was dissolved in alkaline solution (pH 11) and precipitated by rapid addition of strong HNO_3_, dropping the pH down to 2, the average particle size was ca. 85 nm. These particles were more susceptible to pH changes than CLPs precipitated from the ethylene glycol solution. Furthermore, the shape of the CLPs precipitated in this manner was irregular. After these studies, several researchers have utilized the same methodological principles to prepare CLPs (Lievonen et al. 2016; Qian et al. 2014), but novel technologies such as the aerosol flow reactor were developed at the same time (Ago et al. 2016, 2017).

### Smooth colloidal lignin particles

Addition of H_2_O dropwise to acetylated lignin–tetrahydrofuran (THF) solution produced smooth spherical CLPs (Qian et al. 2014). After precipitation, the particles (ca. 80 nm) could be dehydrated using spray, vacuum- or freeze-drying. The degree of lignin acetylation had a significant effect on the shape, uniformity, and concentration of H_2_O needed to initiate CLP formation. Lievonen et al. (2016) prepared CLPs directly from unmodified Kraft lignin using the same solvent system. The smooth, spherical CLPs were stable for months. The effect of the initial lignin concentration on CLP formation had a significant effect on the particle size. When the lignin concentration was 2 mg ml^−1^ the particle size was 320–360 nm and zeta potential ca. – 30 mV. When the concentration was 5 mg ml^−1^ the average particle size was ca. 600 nm the zeta potential being on the same level. Accordingly, Sipponen et al. (2017) prepared positively charged CLPs by adding glycidyltrimethylammonium chloride into lignin solutions, following dissolution of the reaction product in an alkaline solution.

**Fig. 1:**
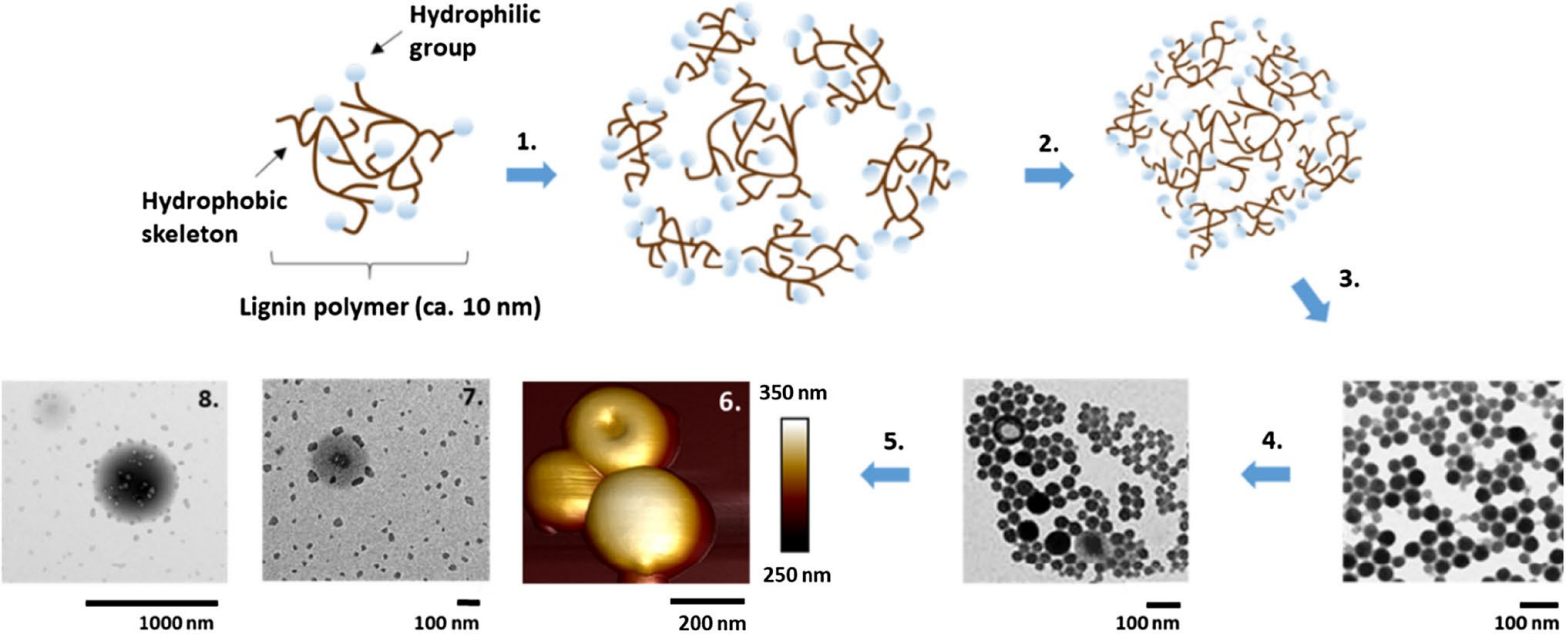
CLP formation using solvent switching based on the polarity difference of the chemicals. *Steps 1*–*3* tiny CLP (ca. 30 nm) formation including clustering of the particles via self-assembling. *Step 4* tiny CLPs colliding with each other forming large aggregates. *Step 5* formation of stable spherical nanosized lignin particles (ca. 200 nm) having smooth polar surface and hydrophobic core. *Step 6* AFM image of the single CLPs, one hollow particle is collapsed. *Steps 7* and *8* TEM images showing growing of the CLPs (Sipponen et al. 2018b; Mattinen et al. 2018a; Qian et al. 2014)

The rational design of nanostructured lignin-based functional materials for medicine, cosmetics, and foods requires a deep understanding of the self-assembly mechanism of CLPs upon solvent exchange or addition of an anti-solvent. The surface fractal structure and stability of CLPs are strongly solvent and pH dependent (Salentinig and Schubert 2017). The precipitation of lignin dissolved in THF occurs when an anti-solvent, often H_2_O, is added, due to the rapid switch of polarity in the solvent system. The mechanism proposed by Qian et al. (2014) argued that the hydrophobic parts of the lignin polymers assimilate together, making the polar groups pointing towards the surface of the particles, yielding an exceptionally hydrophilic surface. Sipponen et al. (2018b) proposed a slightly different mechanism, arguing that the growth of the CLPs begins from the formation of tiny particles by precipitation of large lignin aggregates. When these particles collide, clusters are formed following coating by lignin polymers with exceptionally polar structures. As a result, stable water-dispersible CLPs are produced. Figure 1 shows the scheme for the different steps including the representative atomic force microscope (AFM) and transmission electron microscope (TEM) images.

### Hollow lignin nanoparticles

Hollow CLPs are potential carries for hydrophobic substances to be used in various controlled-release applications. Xiong et al. (2017) and Li et al. (2016) formed particles with high load capacity using enzymatically hydrolyzed lignin (EHL) and Kraft respectively. In the studies Xiong et al. (2017) particles 400–600 nm in diameter were produced by slow addition of H_2_O into solutions of EHL dissolved in THF (0.5–2 mg ml^−1^) following removal of the organic solvent by dialysis. Low initial concentrations of lignin before precipitation yielded CLPs having a large pore radius ca. 60 nm. When using high lignin concentrations, it was only ca. 14 nm. The hollow structure of CLPs resulted from the very slow formation of the particles. When the anti-solvent concentration increased, the hydrophobic interactions between the aromatic groups on the CLP surfaces strengthened leading to the aggregation. Because of the slow change in the polarity, the aggregates entrapped THF during the process forming a nanoemulsion system as an intermediate. When the concentration of H_2_O was high enough, the droplet structure of the emulsion collapsed, and hollow CLPs were formed. The stability of the particles was excellent as no aggregation occurred during the first 15 days, although some aggregation was observed after 40 days. Condensed CLPs were produced when the anti-solvent was quickly added or when the lignin solution was rapidly mixed during the addition of H_2_O. Mishra and Wimmer (2017) used aerosol assisted self-assembly to tailor hollow CLPs.

**Fig. 2:**
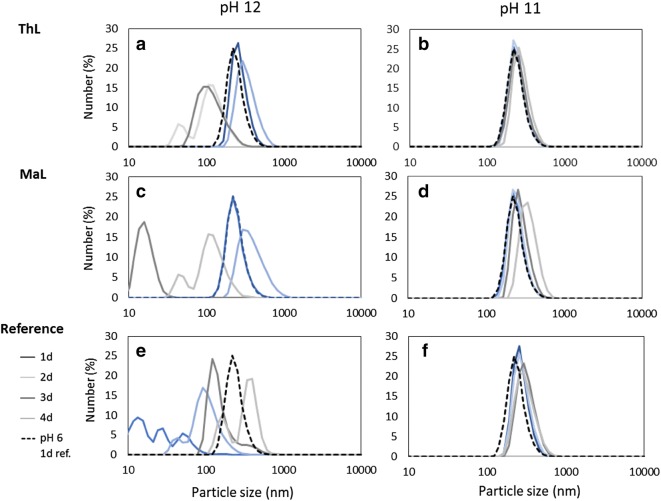
Particle size distributions of CLPs (0.1 mg ml^−1^) prepared from LignoBoost™ Kraft obtained from Domtar Plant (NC, USA) following cross-linking (1000 nkat g^−1^) with ThL (**a**, **b**) and MaL (**c**, **d**) from VTT (Espoo, Finland). The corresponding references are in **e** and **f**. After the enzymatic treatment, the cross-linked CLPs were dried, redispersed in alkali and analyzed using Malvern Zetasizer (Nano-ZS90 instrument, UK). Stability of the dispersions was followed for several days

In many cases, the preparation of tailored CLPs is a straightforward procedure requiring only a small amount of environmentally harmful organic solvents. In general, the stability of CLPs is excellent. Extremely alkaline conditions (Fig. 2) or organic solvents (Mattinen et al. 2018a) are required to dissolve them. When producing nanoparticles in (semi)industrial scale, the organic solvents should be recycled efficiently to keep the process economic and environmentally friendly (Ashok et al. 2018; Lintinen et al. 2018; Leskinen et al. 2019).

## Functionalization and cross-linking of CLPs

Different CLPs are potential additives for various blends, formulations, and matrices varying from industrial bulk to value-added products (Farooq et al. 2019; Pillai et al. 2018; Lintinen et al. 2016). Some tailoring and/or enhancement of the natural properties of lignin are typically required to meet the specific requirements of the applications. Stability, shape, porosity, surface charge and functional groups are common targets for the modifications, achievable e.g. via surface-coating and/or chemo-enzymatic syntheses.

### Coating

Lievonen et al. (2016) coated anionic CLPs from Kraft with positive poly(diallyldimethylammoniumchloride) (PDADMAC). The surface charge of CLPs reversed from − 35 to 30 mV, which was enough for good stability. Furthermore, CLPs have been coated with positively charged proteins such as β-casein (Mattinen et al. 2018b), gelatin and collagen (Leskinen et al. 2017) and cationic lignin (Sipponen et al. 2017) yielding stable CLPs e.g. for adhering protein matrices- and stabilizing Pickering emulsions, respectively. A combination of these two approaches produced effective biocatalyst when lipases were adsorbed onto cationized CLPs following entrapment within alginate beads (Sipponen et al. 2018a). The reactivity of the beads containing enzyme-coated CLPs was verified using butyl butyrate synthesis as an indication reaction. The enzymatic reaction proceeded linearly till 37% during the first 24 h being three times faster than the reference i.e. the alginate beads containing only lipase. In addition to above-mentioned proteins, CLPs could be used as vectors for hormones, peptides, therapeutic proteins, and immunoglobulins as well as antibacterial agents and virus-adherers, for e.g. cosmetics and biomedicine (Henn and Mattinen 2019). Accordingly, Zimniewska et al. (2008) could increase UV-protection and antimicrobial properties of linen using CLP coating.

### Chemical modification

CLPs have shown their usefulness and applicability in multiple industrial sectors, however, the solubility in organic solvents limits their use. Cross-linking is a straightforward method to stabilize their spherical morphology. In addition to heat treatment at high temperatures (Yang et al. 2016a; Patil and Argyropoulos 2017) covalent linkages could be formed via chemical reaction with epichlorohydrin (1-chloro-2,3-epoxypropane) as shown by Nypelö et al. (2015) and Frangville et al. (2012) or using metal–bioorganic sol–gel reactions (Lintinen et al. 2016). However, these cross-linking agents are hazardous in industrial scale applications. Radical polymerization based on the reversible addition–fragmentation chain-transfer (RAFT) mechanism is an excellent method to tailor CLP surfaces with polyacrylamide via covalent linkages (Silmore et al. 2016). Liu et al. (2015) used 2-bromoisobutyrylbromide modified Kraft lignin to prepare poly(2-[diethylamino]ethylmethacrylate) (DEAEMA) grafted CLPs via atom transfer radical polymerization reaction for gene delivery applications. The particle size was 80–130 nm and the surface charge − 45 to – 35 mV.

Unmodified CLPs possess little to no antimicrobial property since the aromatic lignin sidechains are oriented towards inner part of the particles. Richter et al. (2015) produced antimicrobial CLPs by infusing the particles with silver ions. Antimicrobial activity of silver-infused CLPs on *Pseudomonas aeruginosa*, *Escherichia coli*, *Staphylococcus epidermidis* and *Ralstonia* increased significantly compared to non-silver infused CLPs. However, Gilca and Popa (2013) obtained somewhat controversial results. They showed that epoxidated lignin nanoparticles decreased the biodegradation rate of poplar and oak veneer samples. The biodegradation was studied by burying non-treated and particle-immersed samples in garden soil for 6 months. The chemically cross-linked CLPs prepared from Protobind 3000 with high phenolic- and carboxylic acid group content showed the strongest biocidal effect on poplar with a mass loss of ca. 10%. However, the Protobind 1000 derived CLPs containing less carboxylic acid groups had the strongest biocidal effect on oak with a mass loss of ca. 17%. For the non-particle treated references, the mass losses were 37% and 40%, respectively. Cytotoxicity of CLPs for carrier system applications was recently reviewed by Sipponen et al. (2019).

### Enzymatic oxidation

**Fig. 3:**
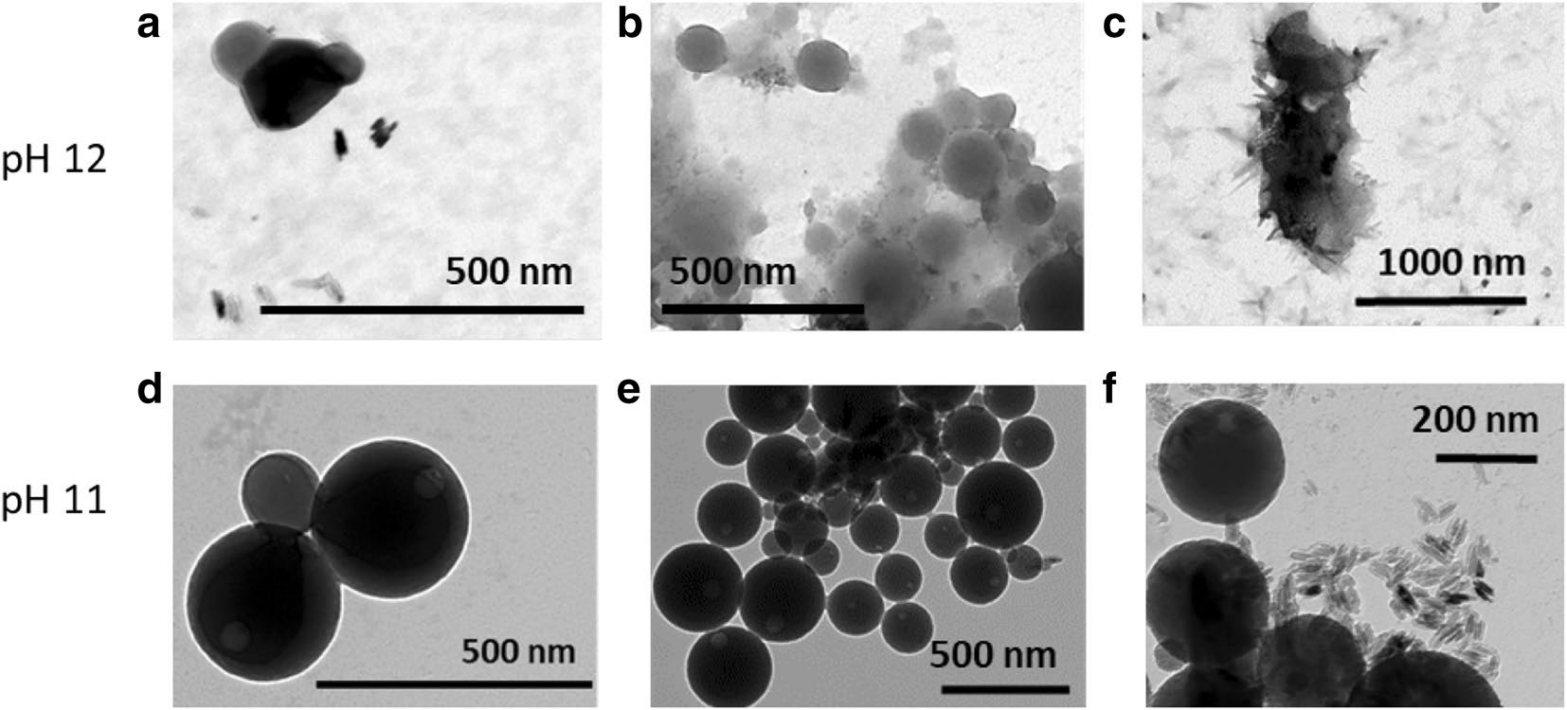
TEM (FEI Tecnai 12 TEM, USA) images of CLPs (0.1 mg ml^−1^) cross-linked (1000 nkat g^−1^) with ThL (**a**, **d**) and MaL (**b**, **e**) after redispersing the particles in alkali at pH 12 and 11, respectively, including the corresponding references (**c**, **f**). The images we obtained 4 days after pH adjustment and particle size measurements

Laccase-catalyzed cross-linking reactions on lignin and lignin model compounds in different solvent systems have been extensively studied to understand the structure–function properties of the enzymes (Cannatelli and Ragauskas 2016; Van de Pas et al. 2011). Recently Mattinen et al. (2018a) showed that fungal laccases such as *Trametes hirsuta* (ThL) and *Melanocarpus albomyces* (MaL) could be used to cross-link CLPs, increasing their stability in organic solvents. Recent studies in alkaline reaction conditions verified these conclusions as in extremely alkaline reaction conditions at pH 12 (Fig. 3a–c), the enzymatically cross-linked CLPs remained stable for several days, but the non-cross-linked CLPs dissolved after 4 days incubation. At pH 11, these particles remained stable (Fig. 3d–f). Cross-linking enzymes such as laccases and *trans*glutaminases forming intra- and intermolecular covalent bonds could be utilized to improve stability and functionality of the non-coated and protein-coated CLPs, respectively (Mattinen et al. 2018a, b). Clearly, the enzymes utilizing food-grade biopolymers as substrates are ecologically and economically viable alternatives to chemical synthetic methods.

## Applications

CLPs are interesting nanobiomaterials to be exploited as green structural components in medical, food, and cosmetic products. These include additives to enhance mechanical properties and/or to give desired functionality for drug and gene carriers, tissue-engineering scaffolds, anchors for biocatalyst, coatings, and emulsifiers (Zeeb et al. 2017; Shin et al. 2017; Sipponen et al. 2018a, b; Rose et al. 2014; Cao and Dobrynin 2016; Mattinen et al. 2018b; Qian et al. 2015).

### Carriers for drug delivery and gene transfer

Hydrophobic drugs and genetic material could be entrapped in CLPs during the formation of the nanoparticles or using post precipitation (Tortora et al. 2014; Gilca et al. 2015; Liu et al. 2015). Chen et al. (2016) produced nanosized lignin capsules by ultrasonicating allyl-grafted lignin in an oil–H_2_O emulsion. The model drug, Coumarin-6, could be encapsulated in the particles by adding the compound into the oil phase before sonicating. During the treatment, capsules were chemically cross-linked on the surface, the hydrophobic drug remaining in the core of the particles. The release of the hydrophobic drug was pH controlled. Also, Figueiredo et al. (2017) studied in vitro applicability of CLPs for drug delivery including enhanced antiproliferation effect in cancer cells. It was shown that unmodified CLPs were non-cytotoxic in general, but that the cytotoxicity depended strongly on the cell type. Drug delivery is clearly a promising application for CLPs, but their potential use as vectors for gene delivery has been studied as well. The nanosized lignin-g-DEAEMA particles prepared according to Liu et al. (2015) showed good DNA condensation via positive DEAEMA sidechains. The transfection efficiency of DNA into cells was studied using a luciferase-encoding gene as a marker in Cos-7, MDA-MB-231, and Hela cell lines. The transfection efficiency was shown to be dependent on the cell-lines, being strongest for the MDA-MB-231. The transfection efficiency decreased, and the cytotoxicity increased with increasing length of DEAEMA-sidechains. It was shown that five to six units were enough for a good transfection.

### Adhesives for wound sealing and tissue engineering

Mattinen et al. (2018b) prepared agglutinative CLPs from lignin and protein side-streams. The gluey properties of different CLPs on soft material were studied using chamois as a model matrix. When using uncoated CLPs for the adhesion, the tensile stress improved ca. 10 and strain ca. 6 times compared to that of polymeric Kraft lignin. For the β-casein nanoparticles, the improvements were 20 and 8 times, respectively. The enhancement in tensile strain and stress for the β-casein coated CLPs remained between those of CLPs and β-casein particles. The adhesiveness of CLPs rests on the ability of nanosized particles, having a large surface area, to penetrate and interact via bridging mechanism with polymeric chains on the soft surface (Rose et al. 2014; Cao and Dobrynin 2016). Coating CLPs with β-casein clearly improved the non-covalent interactions with the protein matrix. Furthermore, the agglutinations could be strengthened using transglutaminase for fast curing. In addition to protein coating, oxidative enzymes such as laccases could be used to modify the polarity of the CLP surfaces (Van de Pas et al. 2011). Regarding to the medical applications, enzymatic reactions are fast and feasible in moist environments. Furthermore, nanosized aromatic lignin particles enable clinical fluorescence imaging that could be utilized for following reactions and the lifetime of CLPs in the living human body (Shin et al. 2017; Pillai et al. 2018).

### Food additives

The effect of CLPs as an additive to tailor multifunctional polymer films for food packaging was studied by Yang et al. (2016b). To obtain coatings with antioxidant and -microbial properties, polyvinyl alcohol (PVA) and chitosan films were modified with CLPs. When the quantity of CLPs in the PVA blend was 1–3 wt%, the mechanical and thermal properties of the films improved, along with gaining UV-shielding and antimicrobial properties. The chitosan films became tougher with improved elongation at the break down point. In addition, the researchers showed (Yang et al. 2015) that the properties of biodegradable wheat gluten films could be enhanced by including CLPs in the composite structure. Accordingly, Farooq et al. (2019) prepared composite films from cellulose nanofibrils (CNFs). Addition of CLPs enhanced the water resistance and mechanical properties of the fibrils significantly along with the improved UV-shielding and antioxidant properties.

## Conclusions

The need to search for the alternatives for the fossil-based products combined with the increased awareness of the negative impacts of these goods on the environment is enormous. Environmentally friendly and biodegradable CLPs could be utilized to improve existing products by enhancing e.g. mechanical strength, water, gas and UV-resistance of the materials to reach requirements of the practical use. CLPs could be prepared by simply dissolving lignin in organic solvents following precipitation by addition of an anti-solvent. These particles could be easily tailored e.g. covalently via cross-linking or non-covalently via surface adhesion or infusion of various substances. The most prominent applications of CLPs in medicine, food and cosmetic sectors are listed in Table 1. Furthermore, these particles could be exploited in industrial flotation processes and in bioprocessing e.g. to enhance enzyme activities. Smart multi-functional barriers such as self-cleaning hydrophobic surfaces and photocatalytic coatings could be prepared for more technical use. Hence, the most likely applications to be first realized in full industrial scale use include different coatings and adhesives. In order to enable this, scale-up of the CLP preparation processes following large scale feasibility tests are required. The increased breakthrough research and the number of potential applications of CLPs indicate that the future for the utilization of technical lignin in various forms is very optimistic.

**Table 1:** Summary of the value-added applications of CLPs along with the selected references

Applications	Characteristics	References
Medicaments		
Pharmaceuticals	Drug and gene carriers, wound sealing materials, adhesives, hydrogels, surfactants	Chen et al. (2016), Kai et al. (2016), Ago et al. (2016), Figueiredo et al. (2017, 2018), Liu et al. (2015), Tardy et al. (2018), and Silmore et al. (2016)
Tissue engineering		
Pickering emulsions		
Foods and packings		
Processing	Biocatalysts, structure engineering, edible coatings, adsorbents, fillers, anti-oxidative and -microbial agents, stabilizers	Sipponen et al. (2017, 2018a), Farooq et al. (2019), Cannatelli and Ragauskas (2016), Mattinen et al. (2018a, b), Duval and Lawoko (2014), Yang et al. (2015, 2016b) and Richter et al. (2015)
Coatings		
Emulsifiers		
Cosmetics		
Sunscreens	Antimicrobial agents, UV-protectors, stabilizers	Qiu et al. (2016), Frangville et al. (2012) and Mishra and Wimmer (2017)
Creams		
